# Interactions of Alphavirus nsP3 Protein with Host Proteins

**DOI:** 10.3389/fmicb.2017.02652

**Published:** 2018-01-09

**Authors:** Tyler Lark, Forrest Keck, Aarthi Narayanan

**Affiliations:** National Center for Biodefense and Infectious Diseases, School of Systems Biology, George Mason University, Fairfax, VA, United States

**Keywords:** alphavirus, nsP3, stress granules, DDX1/DDX3, IKKβ, heat shock proteins, PI3K-Akt-mTOR, poly ADP

## Abstract

Alphaviruses are members of the *Togaviridae* family and are grouped into two categories: arthritogenic and encephalitic. Arthritogenic alphavirus infections, as the name implies, are associated with arthritic outcomes while encephalitic alphavirus infections can lead to encephalitic outcomes in the infected host. Of the non-structural proteins (nsPs) that the viruses code for, nsP3 is the least understood in terms of function. Alphavirus nsP3s are characterized by regions with significantly conserved domain structure along with regions of high variability. Interactions of nsP3 with several host proteins have been documented including, stress granule-related proteins, dead box proteins, heat shock proteins, and kinases. In some cases, in addition to the interaction, requirement of the interaction to support infection has been demonstrated. An understanding of the proteomic network of nsP3 and the mechanisms by which these interactions support the establishment of a productive infection would make alphavirus nsP3 an interesting target for design of effective medical countermeasures.

## Introduction

Alphaviruses belong to the family *Togaviridae* and are divided into two categories: arthritogenic and encephalitic alphaviruses (Strauss and Strauss, 1994). The classification is indicative of their geographic locale and differences in pathogenesis and symptoms, namely New World alphaviruses tend to be associated with encephalitic phenotypes in the affected host while Old World alphaviruses are more likely to cause arthritic outcomes (Fros and Pijlman, 2016).

Alphaviruses are spherical in shape, ∼65 nm in diameter, and enveloped with an icosahedral capsid (Jose et al., 2009). The genome consists of an ∼11.5-kb positive sense, single-stranded RNA with a 5′ cap and 3′ poly(A) tail (Garmashova et al., 2007), which encodes five structural (capsid, E1–E3, 6K) and four non-structural proteins (nsPs: nsP1–4). With regard to functionality associated with structural proteins, capsid binds to viral RNA and stimulates nucleocapsid assembly and RNA synthesis; E1 stimulates the fusion of the viral membrane with the endosomal membrane; E2 aids in the binding to the target receptor and viral uptake via receptor-mediated endocytosis (Dubuisson and Rice, 1993); E3 aids in the folding of the precursor E2 protein and in the activation of E1 (Lobigs et al., 1990); 6K forms ion channels within the host cell and aids in trafficking, assembly (Lusa et al., 1991), and budding of the virus. As for the nsPs, nsP1 is a membrane-anchored protein that is responsible for the synthesis and capping of RNA (Wang et al., 1991); nsP2 acts as a RNA helicase (Gomez de Cedrón et al., 1999) and cleaves the non-structural polyprotein into its individual elements (Strauss et al., 1992); nsP4 acts as an RNA polymerase (Hahn et al., 1989). Little is known about the function of nsP3 other than an appreciation of its requirement for RNA synthesis (Strauss and Strauss, 1994; Garmashova et al., 2007; Fros and Pijlman, 2016). This review will elucidate the interactions of nsP3 with host proteins in order to better understand its role in the life cycle of alphaviruses. Where possible, differences in protein interactions between arthritogenic and encephalitic alphaviruses will be highlighted.

## nsP3 Structure

**FIGURE 1:**
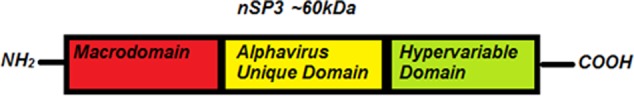
Schematic diagram of nsP3. The nsP3 protein consists of three regions: macrodomain, alphavirus unique domain, and hypervariable domain. The macrodomain is highly conserved among all alphaviruses and contains the ADP-ribose-binding site. The alphavirus unique domain contains many serine and threonine residues and is the location of the zinc-binding site. The hypervariable domain comprises the final third of the protein at the C-terminal, containing many sites for phosphorylation. This region varies greatly among the alphaviruses in both length and sequence.

The nsP3 is an ∼60 kDa-sized protein that can be divided into three different domains: macrodomain, alphavirus unique domain, and hypervariable region (**Figure 1**; Rupp et al., 2015). The macrodomain is located on the N-terminus of the protein and is a highly conserved sequence of ∼150 amino acids, which is also found in other viruses such as coronaviruses (Beitzel et al., 2010; Rungrotmongkol et al., 2010). The structure of the this domain consists of a six-stranded, twisting, centrally located β-sheet that is surrounded by four α-helices, three on one side and one on the other. This β-sheet, α-helix arrangement is preserved among both arthritogenic and encephalitic alphaviruses. Within this region, there is a crevice for the interaction with adenosine diphosphate ribose (ADP ribose) (Karras et al., 2005; Malet et al., 2009; Park and Griffin, 2009a; Foy et al., 2013), whose role will be expounded upon in a later section. The alphavirus unique domain comprises the middle third of nsP3 and is maintained only within alphaviruses (Rupp et al., 2015). This segment of the protein contains many serine and threonine residues, forming two parallel β-sheets and antiparallel α-helices. This domain also includes a zinc-binding domain which is important in cleaving of the P2/3 polyprotein, infectivity, and RNA production (Shin et al., 2012; Rupp et al., 2015). The hypervariable region is located on the C-terminus of the protein. Unlike the macrodomain and alphavirus unique domain, the hypervariable domain is not conserved among alphaviruses in sequence or structure. This segment is rich in serine and threonine residues and offers numerous sites for phosphorylation (Foy et al., 2013). The length of this region can vary based on the alphavirus, and even those which have a similar length, such as Venezuelan Equine Encephalitis Virus (VEEV) and Sindbis Virus (SINV), differ in the sequence of amino acids that make up this section. This variability could account for, or at least play a role, in the difference in pathogenicity and protein interactions of nsP3 in the context of different viruses (Varjak et al., 2010). However, it has been noted that there is a certain level of conservation among several alphaviruses in the C-terminal domain. Removal of this conserved sequence area in Semliki Forest Virus (SFV) showed a decrease in infectivity of the virus, attesting to its importance in the infectious process (Vihinen et al., 2001).

## nsP3 Interactions

### ADP-Ribose-Binding Site

The ADP-ribose-binding site of alphaviruses is conserved in structure, but amino acid residues may differ between arthritogenic and encephalitic viruses. The ability to bind ADP ribose, as well as RNA, was shown to be comparable between VEEV and Chikungunya Viruses (CHIKV) (Malet et al., 2009), although VEEV has stronger affinity than CHIKV (Park and Griffin, 2009b). Activity as an ADP-ribose 1″-phosphate phosphatase activity was similar between the two viruses. However, SFV phosphatase activity was detected at a much lower level (Malet et al., 2009), suggesting that differences in activity may vary from species to species rather than being able to group by phosphatase activity solely on arthritogenic and encephalitic classification. There is also no correlation in ability to bind monomeric ADP ribose and poly ADP ribose. Studies with SFV and Hepatitis E virus, another macrodomain containing virus, show that both effectively bind poly ADP ribose, but Hepatitis E virus has an affinity for monomeric ADP ribose while SFV has no attraction (Neuvonen and Ahola, 2009). The role of binding to poly or monomeric ADP ribose in the life cycle of the viruses is unclear. For CHIKV, the ability to hydrolyze monomeric ADP ribose was shown to be essential for both viral replication and virulence. Mutant nsP3 viruses that lacked the ability to either bind or hydrolyze ADP ribose had a significant decrease in viral titers in *in situ* experiments. *In vivo* studies revealed that hindrance of both binding and hydrolyzing capabilities greatly reduced virulence in mice, but interestingly, hydrolyzing mutants maintained virulence (McPherson et al., 2017). In eukaryotic cells, monomeric ADP ribosylation influences processes such as immune response and metabolism, and poly ADP ribosylation is an important post-translational modification event. Poly ADP-ribose polymerases (PARP) facilitate this event. There are a wide number of PARPs and they respond to different stimuli within the cell. PARP-1 is stimulated by damage to cellular DNA and is a key factor in the survival of the cell (Schreiber et al., 2006). When mutations were made in the ADP-ribose-binding site of nsP3 of SINV, there was no effect on the virus’ ability to bind poly ADP ribose in mouse neuronal cells. However, the virus’ ability to replicate was hindered. The ability of the virus to form replication complexes and synthesize RNA was greatly diminished. An increase in neuronal cell death was higher in these mutated nsP3 viruses as compared to the wild type (Malet et al., 2009). Further investigation into this showed that PARP-1 is closely associated with nsP3 functionality. Co-immunoprecipitation of PARP-1 in infected cells revealed that the polymerase was associated with nsP3 in the replication complex. The other nsPs are not required for association of PARP-1 with nsP3, and enzymatic activity did not affect viral replication. Surprisingly, PARP-1 was not associating with the ADP-ribose-binding site, but rather with the C-terminal domain. It is thought that this recruitment of PARP-1 is important in stabilizing the replication complex or for the recruitment of other host proteins (Park and Griffin, 2009a).

### Stress Granule-Related Proteins

During cellular stress, the translation of mRNA can be interrupted, leading to their cytoplasmic accumulation. This cytoplasmic accumulation of mRNPs and stress proteins results in the formation of stress granules. The function of these complexes is either to carry out the unfinished translation of the mRNA or to degrade the molecule (Buchan and Parker, 2009). Alphavirus nsP3 has been documented as interacting with proteins related to stress granule formation. There are differences in how arthritogenic and encephalitic alphavirus nsP3 proteins interact with components of the stress granules. Arthritogenic alphavirus nsP3 interacts with the host protein Ras-GTPase activating SH3-binding domain protein (G3BP). G3BPs are RNA-binding proteins that aid in the organization of the stress granule. Arthritogenic nsP3 interacts with G3BPs through a region on the C-terminal domain containing two FGDF motifs, two phenylalanine residues which are separated by a glycine and aspartate residue. This area was found to be conserved in multiple arthritogenic viruses, suggesting that they use the same mechanism to recruit G3BPs. Most other FGDF-containing proteins contain only a single motif as compared to the two found in the alphavirus. Having two may ensure the rapid sequestering of G3BP before stress granules can be formed (Panas et al., 2014, 2015; Schulte et al., 2016). The interaction of nsP3 and G3BP has been extensively studied in SINV, CHIKV, and SFV. G3BP1 and G3BP2 are found spread throughout the cell, and soon after infection those are localized to nsP3 containing replication complexes in SINV infections (Frolova et al., 2006; Gorchakov et al., 2008). Studies with CHIKV show that this attraction leads to the formation of stress granule-like complexes that differ in composition to true stress granules and are non-functional. During later parts of replication, G3BP is shifted away from the replication complex associated with the cell’s membrane toward the cytoplasm. Interestingly, it was found that reducing the level of G3BP1 and G3BP2 resulted in a decrease in the amount of viral RNA. There was a detectable level of the two proteins associated with the viral replication complex at the cell membrane and was noted to be involved in the switch from translation to minus-strand RNA synthesis (Cristea et al., 2006; Fros et al., 2012; Scholte et al., 2015; Fros and Pijlman, 2016; Kim et al., 2016). This finding is contradictory to another study with SINV that found depleting G3BP1 and G3BP2 levels led to an increase in viral RNA production (Cristea et al., 2010). In SFV infections, the G3BP proteins were also associated with the replication complex early on in infection (Panas et al., 2012). Mosquitoes are natural vectors for the transmission of alphaviruses. Mosquito cells and live mosquitoes, *Aedes albopictus*, were infected with CHIKV to determine whether a similar association with stress granules existed. The mosquito G3BP homolog, Rasputin, was found to be localized to nsP3 foci. Silencing of Rasputin in *in vivo* oral infections lead to a reduction in viral production, whereas the same silencing in *in vitro* studies did not affect viral replication. It has been proposed that interaction between nsP3 and Rasputin may interfere with immune responses (Fros et al., 2015).

Whereas arthritogenic alphavirus nsP3 has been shown to interact with G3BP proteins, encephalitic alphavirus nsP3 did not co-localize with the protein. VEEV forms nsP3-associated complexes, but the hypervariable domain was shown to not bind to G3BP. Replacing the hypervariable domain of SINV with that of VEEV disrupted the virus’ ability to associate with G3BP (Foy et al., 2013). Rather, it was shown that VEEV associates with Fragile-X-related (FXR) proteins. FXR exists in two forms, FXR1 and FXR2, which are homologs of Fragile-X mental retardation protein. These proteins formed heterodimers and associated with mRNPs in the formation of stress granules (Tamanini et al., 1999, 2000; Anderson and Kedersha, 2008). Inhibition of both FXR proteins reduced the level of virus being produced, and inhibition of FMRP exacerbated the effect. The binding of nsP3 to the Fragile-X proteins promoted the formation of the viral replication complex and RNA replication. There was also an accumulation of viral G RNA in these complexes, which was needed for translation of the early polyproteins (Kim et al., 2016).

Interestingly, Eastern Equine Encephalitis Virus (EEEV) has recently been shown to be capable of utilizing both FXR and G3BP proteins. The HVD of EEEV contains a single-binding site for both sets of proteins and the binding of one is independent of the other. As a result of this independent binding, inhibition of only one of the proteins did not result in a decrease in viral replication and dual inhibition was critical for inhibition. It was suggested that binding to both FXR and G3BP proteins played a role in the high pathogenicity of EEEV and may show the evolutionary branching of the HVD from the G3BP binding of the arthritogenic alphaviruses and the FXR binding of encephalitic alphaviruses (Frolov et al., 2017).

### Amphiphysins

Amphiphysins are a family of proteins that are involved in various cellular processes such as endocytosis and membrane trafficking. Amphiphysin 1 and 2 are predominately found in neuronal cells and are crucial for the endocytosis of clathrin-mediated vesicles in synapses. They facilitate this process through an interaction with dynamin via their SH3 domain (Wigge and McMahon, 1998). The SH3 domain is a non-catalytic domain that is found on numerous signaling proteins (Weng et al., 1995). Alphaviruses are able to interact with this SH3 domain to recruit amphiphysin to the site of replication complexes (Neuvonen et al., 2011). While nsP3 interacts with the same SH3-binding site as dynamin, it does so with a higher affinity (Tossavainen et al., 2016). Infections with SFV and SINV showed that during early time points amphiphysin 1 localized to the replication complex and to the plasma membrane in later time points. Amphiphysin 2 localized in a similar pattern at early time points, but in later time points, it was observed in cytopathic vacuoles. This difference in localization at the different time points could be attributed to the differences in functionalities of the two proteins. Inhibiting this localization through mutations in the SH3 domain was shown to inhibit the production of viral RNA (Neuvonen et al., 2011). Their importance in viral replication could be linked to the association of the alphavirus replication complex to membranes. The replication complex is initially formed on the plasma membrane after entry and is transported to early endosomes and lastly ends up in invaginations of the lysosomal membrane. nsP3 is located on the surface of these spherules in order to interact with other proteins (Froshauer et al., 1988; Peränen and Kääriäinen, 1991; Jürchott et al., 2003; Spuul et al., 2011). The involvement of amphiphysins in endocytosis and membrane trafficking may suggest that they are important in the formation of this invagination of lysosomal membranes to house the viral replication complex.

### Y-Box-Binding Protein 1 (YBX1)

Y-box-binding protein 1 (YBX1) was shown to be associated with nsP3 of SINV. YBX1 was coupled to nsP3 in complexes located on endosomal membranes as well as in the nucleus (Gorchakov et al., 2008). However, the importance of this interaction has not been elucidated. YBX1 is capable of shuttling between the cytoplasm and the nucleus and has been implicated in numerous cellular functions. In the nucleus, it controls the transitioning of the cell cycle from G1 to S phase (Jürchott et al., 2003), DNA replication and repair, and transcriptional activation. In the cytoplasm, YBX1 aids in chaperoning and translation of mRNA and is a component of mRNPs (Matsumoto and Wolffe, 1998; Lu et al., 2005; Eliseeva et al., 2011). YBX1 is organized as three domains: a variable N-terminal domain, cold shock domain, and C-terminal domain. The cold shock and C-terminal domain are capable of binding ssRNA, ssDNA, DNA, and proteins (Kloks et al., 2002; Mihailovich et al., 2010). Looking at YBX1 interactions with other viruses does not clear the picture as to its role in binding to alphavirus nsP3. In Dengue virus infections, YBX1 inhibits the production of viral particles. In this association, YBX1 binds to the 3′ untranslated region and suppresses translation of the viral RNA (Paranjape and Harris, 2007). It remains unclear whether the lack of a poly(A) tail in Dengue virus could explain differential utility of YBX1 in this case as compared to alphaviruses. However, when looking at Hepatitis C virus, YBX1 acts as both a stimulant and a repressor. The association of YBX1 with NS3/4a is necessary for an efficient replication of viral RNA. There is an inverse correlation between the number of viral particles produced and the amount of YBX1 present. This shows that YBX1 is an important regulator in controlling Hepatitis C replication and virus assembly (Chatel-Chaix et al., 2011). The human polyomavirus JC virus makes use of YBX1 as a transcriptional activator to facilitate its survival (Raj et al., 1996). YBX1 has been shown to aid in cell survival during stress (Lu et al., 2005) and inhibits MHCII molecules (Didier et al., 1988; Ting et al., 1994). With a prominent nuclear phase involved in polyomavirus life cycle, the role of YBX1 can have different implications as an enabler of viral transcription as contrasted with alphaviruses where the life cycle is predominantly cytoplasmic. Taking these into account, it is possible that YBX1 may play a role in the replication of alphaviruses or be involved in keeping the cell alive by suppressing immune responses. More studies need to be done to determine the functional significance of the YBX1:nsP3 interaction in alphavirus life cycle.

### Heat Shock Proteins

Heat shock proteins are regulatory proteins that play essential roles during cellular stress. They act as chaperones and maintain proper folding of proteins that would otherwise denature during stress responses. There are multiple members of the heat shock protein family whose function can be both tissue and organelle specific (Jee, 2016). The Hsp70 and Hsp90 family have been show to play a role in the life cycle of alphaviruses through nsP3 interactions. The Hsp70 family consists mainly of two proteins: Hsc70 and Hsp70. Hsc70 is a constitutively expressed heat shock cognate protein. It functions in aiding in the uncoating of clathrin-coated vesicles, transporting proteins to various organelles, and targeting proteins to lysosomes. Hsp70, unlike Hsc70, is only expressed during stress conditions. The interactions that Hsp70 has are very similar to Hsc70, with only a few differences, such as associating with immunogenic peptides (Goldfarb et al., 2006; Liu et al., 2012). A study looking at host protein associations with SINV nsP3 showed an interaction with Hsc70 (Gorchakov et al., 2008). The functional relevance of the interaction has yet to be determined, but looking at other viruses shows that Hsc70 is important for the life cycle of viruses. Rotaviruses interact with Hsc70 at the cell surface after binding to their receptor. This interaction mediates the entry of the virus into the cytoplasm of the cell (Zárate et al., 2003). The Hepatitis B virus reverse transcriptase is normally activated by Hsp90, but studies have shown that Hsc70 is also able to act as an enzymatic catalyst to trigger the reverse transcriptase (Beck and Nassal, 2003). In HIV, Hsc70 and Hsp70 aid in the transport of viral polyproteins to the plasma membrane (Gurer et al., 2002). Tombusviruses utilize Hsp70 to insert the viral replicase into the intracellular membranes and efficiently replicate (Serva and Nagy, 2006; Wang et al., 2009). An interaction with the SINV nsP3 for viral entry seems unlikely, as the protein is not expressed on the surface of the virion, but a role in viral replication could be plausible.

Hsp90 aids in the folding and activation of numerous signaling proteins involved in cellular proliferation and survival, such as src family kinases and Raf proteins (Verma et al., 2016). Hsp90 has been shown to interact with the nsP3 of CHIKV to promote viral replication. Hsp90β was co-immunoprecipitated with nsP3 during infections, another Hsp90 was shown to interact with nsp4. Inhibition of Hsp90 was shown to decrease viral RNA and protein production (Rathore et al., 2014). A follow-up study revealed that Hsp90 aids in replication by stabilizing the nsP2 protein (Das et al., 2014). Hsp90 is essential for replication in other viruses as well, aiding in viral RNA polymerase synthesis in flock house virus (Castorena et al., 2007) and stabilizes the L protein of the viral polymerase for vesicular stomatitis virus (Connor et al., 2007). Whether similar functionalities are associated with the alphavirus nsP3 interaction remain to be determined.

### PI3K-Akt-mTOR

The PI3K-Akt-mTOR pathway plays a crucial role in various cellular processes such as cell growth and survival. Phosphatidylinositol-3 kinases (PI3K) is a family of kinases that phosphorylate phosphoinositides to generate phoshatidylinositol-3,4,5-trisphosphate (PIP3). PI3Ks are activated by the binding of a tyrosine kinase receptor (Owonikoko and Khuri, 2013). PIP3 binds to and activates Akt, which is also phosphorylated by PDK1 (Vara et al., 2004). Akt is then able to activate mammalian target of rapamycin (mTOR), which is a regulator of protein synthesis and cell survival (Memmott and Dennis, 2009). The PI3K-Akt-mTOR pathway has been shown to be exploited by numerous viruses, normally to prevent an anti-apoptotic response (Dunn and Connor, 2012; Diehl and Schaal, 2013). nsP3 of SFV was shown to activate Akt directly, but only when associated with the plasma membrane. The hyperphosphorylated region of the HVD region of nsP3 was indicated as being critical for the interaction (Thaa et al., 2015). Activation of this complex was shown to be necessary for the internalization of the replication complex that assembled at the plasma membrane (Spuul et al., 2010). Wild type nsP3 of SFV was able to directly activate Akt, while activation of Akt in SFVΔ50, a mutant strain with a deletion of residues 319–368 in nsP3 hyperphosphorylated region, was suppressed. Interestingly, CHIKV infection only moderately activated Akt and was dependent upon PI3K activation of Akt. The activation of Akt by PI3K did not stimulate the internalization of replication complexes for CHIKV (Thaa et al., 2015). In arthropods, SINV infection led to the activation of the PI3K-Akt-TOR pathway which contributed to an uptick in translation through TOR activation of 4E-BP1, which activates eIF4E, a protein necessary for cap-dependent translation. Translation of both viral and host mRNA is promoted through this activation. Activation of 4E-BP1 is not evident in vertebrates, as alphaviruses can inhibit host translation during infection (Patel and Hardy, 2012). In *in vitro* studies using human cells, SINV was shown to suppress PI3K-Akt-mTOR late in infection. Viral replication was not inhibited by using inhibitors of PI3K and mTOR (Mohankumar et al., 2011). Taken together, these data suggest that the PI3K-Akt-mTOR pathway may not be necessary for the production of virions, but is required for the internalization of the replication complex.

### DDX1/DDX3

DEAD box proteins are a family of ATP-dependent RNA helicases that function in multiple steps of RNA metabolism. Along with the involvement in cellular processes with RNA, DEAD box proteins have also been shown to play a role in antiviral immune response. DDX1 is involved in the sensing of dsRNA and production of an interferon response. Also, it binds to the p65 subunit of NFκB to enhance inflammatory cytokine production (Fullam and Schröder, 2013). DDX3 also has antiviral properties. DDX3 can recognize viral RNA and generate a response through the RIG-I-like receptor family to generate type I IFN. DDX3 binds to IKK𝜀, which phosphorylates DDX3 and recruits IRF3 (Valiente-Echeverría et al., 2015). Phosphorylation of DDX3 by TBK1 promotes the recruitment of DDX3 to the interferon β promoter and induction of interferon production. DDX3 has also been shown to sense viral dsDNA and promote generation of interferon (Ariumi, 2014). Using a mass spectrometry approach, VEEV nsP3 was shown to associate with both DDX1 and DDX3. A knockdown of both helicases resulted in a drop in viral replication. It has been suggested that DDX1 and DDX3 may play a role in the unwinding of viral RNA for replication and/or translation in stress granules (Amaya et al., 2016). DDX1 and DDX3 have been shown to be exploited by numerous other viruses. DDX1 promotes replication in Coronaviruses (Xu et al., 2010) and transcription in JC virus (Sunden et al., 2007). In HIV-1, DDX3 aids in the shuttling of Rev between the nucleus and the cytoplasm and DDX1 acts as a co-factor for the function of Rev (Li et al., 2005). DDX3 is used to stimulate the replication of Hepatitis C virus (Oshiumi et al., 2010; Wang and Ryu, 2010) and is sequestered in Hepatitis B infections to prevent an immune response (Ariumi et al., 2007). An interesting functionality associated with DDX3 as a shuttling protein in the context of alphavirus infectious cycle will be to evaluate whether it has any role to play in the nuclear localization of the capsid protein, and hence influence control of transcription.

### IKKβ

NFκB is a transcription factor that is involved in the activation of genes participating in immune and inflammatory responses, cell proliferation, and survival (Israël, 2010). NFκB is activated in two different manners: a canonical and alternative pathway. Focusing on the canonical pathway, activation begins with the binding of proinflammatory, IL-1 and TNFα, to a Toll-like receptor (Lawrence, 2009). This leads to the recruitment of adaptor proteins, such as TNF receptor associated factors, to phosphorylate the IKK complex. The IKK complex consists of the three subunits IKKα, IKKβ, and IKKγ (Amaya et al., 2014). IKKβ phosphorylates the inhibitory κB proteins associated with the p65/p50 subunits of NFκB. IκBα is then tagged for ubiquitination, releasing the two NFκB subunits for nuclear transportation (Lawrence, 2009). The NFκB pathway is an often-utilized pathway for viruses, such as HTLV-1 (Hai et al., 2006; Hiscott et al., 2006) and Hepatitis B virus (Santoro et al., 2003). A recent study has shown that VEEV nsP3 associated with the IKKβ subunit of the IKK complex, potentially through the HVD region to activate the canonical pathway of NFκB. Viral replication triggered the activation of TLR3, activating the IKKβ complex. Inhibition of IKKβ function led to a broad spectrum decrease in the replication of encephalitic alphavirus in cell culture models and VEEV, *in vivo* (Amaya et al., 2014).

### Applications

Alphavirus nsP3 interactions with host proteins have been demonstrated to play an integral part in the viral replication process. In several of these documented interactions, while descriptive features of the interactions may be available, mechanistic details of functional requirements of such interactions remain to be elucidated. While inferences can be made by looking at other viruses that utilize the same proteins and initial hypotheses of possible functions as related to the establishment of a productive infection can be proposed, it is important to understand that many of these viruses differ greatly from alphaviruses in genomic makeup and life cycle, raising the possibility that the purpose and mechanism of those interactions may be different. As elaborated in several instances above, the same host protein may play opposing roles in the context of different viruses, thus underscoring the very important need to understand how such host proteins influence an infectious cycle within the context of viral biology. Many such contrasting functions may also be manifestations of not just the simple interaction between the target viral and host proteins, but involve larger dynamic protein networks. These multiple interactions of nsP3 and critical importance of the host protein interaction networks offer the additional potential to diversify the target portfolio for development of therapeutic candidates and identification of candidates that are supportive of broad spectrum inhibition. Of relevance, the inhibition of many of these interactions has been shown to impede viral replication. Use of RK-33, a small molecule inhibitor of DDX3, decreased infectious viral titers for VEEV (Amaya et al., 2016). The use of an IKKβ inhibitor had a negative effect on the ability of VEEV to efficiently replicate (Amaya et al., 2014). Cell lines with a knockout of FXR inhibited VEEV replication, with a similar effect noticed for SINV and CHIKV in cells with knockouts of G3BP1 and G3BP2 (Kim et al., 2016). nsP3 is an important contributor to the virulence of alphaviruses. Multiple studies have shown that creating mutations or deletions within the protein hinders the virus’s ability to effectively replicate in the host. In SFV, deletions in the C-terminal region hampered the production of subgenomic RNA and establishment of infection, results (Varjak et al., 2010) which were also a product of experiments with mutations and deletions in the hypervariable region (Galbraith et al., 2006). Mutations in the hypervariable region also resulted in a virulence in mice inoculated both intranasally and intramuscularly. Mutations inserted into the nsP3 of SINV show a similar result, reduction in viral RNA being produced (LaStarza et al., 1994; Dé et al., 2003). The use of massive parallel sequencing in VEEV has allowed for the identification of regions within the nsP3 that are tolerant of mutations, the C-terminal region, and those that are intolerant of mutations, the N-terminal region (Beitzel et al., 2010). Given the significance that nsP3 has in fostering a productive viral infection, more studies need to be performed to determine the exact mechanisms of interactions nsP3 has with host proteins, elucidate protein interaction networks as variables of stages of viral infectious cycle, and understand their relevance in the establishment of a productive infection.

## Author Contributions

TL, FK, and AN wrote the article. TL was responsible for collation of information regarding nsP3 interaction partners. FK assisted with article preparation and formatting, and in addition to contributing to the written body. AN worked with TL and FK to compose the article and final review.

## Conflict of Interest Statement

The authors declare that the research was conducted in the absence of any commercial or financial relationships that could be construed as a potential conflict of interest.
